# Rapid clinical response in refractory discoid lupus erythematosus treated with anifrolumab^؆^

**DOI:** 10.1016/j.abd.2025.501284

**Published:** 2026-01-14

**Authors:** José Ramos, Hugo Leme, António Magarreiro-Silva

**Affiliations:** Department of Dermatology and Venereology, Unidade Local de Saúde Almada-Seixal, Almada, Portugal

Dear Editor,

Cutaneous Lupus Erythematosus (CLE) is an autoimmune disease with variable clinical expression. It may present as an exclusively cutaneous condition or as one of the multiple manifestations of Systemic Lupus Erythematosus (SLE). Discoid Lupus Erythematosus (DLE) is the most common form of chronic cutaneous lupus erythematosus. Lesions typically affect photoexposed areas and appear as indurated erythematous plaques that evolve with follicular hyperkeratosis, atrophy, and central hypopigmentation with peripheral hyperpigmentation. These lesions can lead to severe and disfiguring scarring, particularly when located on the nose, ear pinnae, and eyelids.^1^

Anifrolumab is a fully human monoclonal antibody targeting the type I interferon receptor subunit.^1^ It was recently approved for the treatment of moderate-to-severe SLE and is administered as a 300 mg intravenous infusion every four weeks. Although it is not currently approved for the treatment of CLE, data from phase III clinical trials (TULIP-1 and TULIP-2) have shown significant improvement in CLE manifestations in patients with SLE.^2^ Additionally, several case reports have demonstrated its rapid and marked efficacy in DLE.^3^, ^4^, ^5^

We report a case of severe, treatment-resistant discoid lupus erythematosus with facial involvement that showed a dramatic and rapid response to anifrolumab.

A 56-year-old woman with Fitzpatrick skin phototype IV and history of smoking presented with longstanding DLE involving the face, auricular region, and forearms. She had been followed in Dermatology for over 10 years and had previously been evaluated for systemic involvement, which was excluded on multiple occasions. Her past treatments included high-potency topical corticosteroids, calcineurin inhibitors, oral prednisolone, and hydroxychloroquine, with inadequate disease control.

After a two-year loss to follow-up, she was re-evaluated. She had continued hydroxychloroquine 400 mg/day and topical corticosteroids but reported no improvement. Clinical examination revealed erythematous-crusted plaques with central hypopigmentation and peripheral hyperpigmentation, atrophy, and signs of chronic inflammation, predominantly affecting the malar region, nasal pyramid, and upper lip. Additional lesions were present on the auricular conchae and forearms. Systemic lupus erythematosus was again excluded. Methotrexate was initiated at 10 mg/week and increased to 20 mg/week, along with strict photoprotection. After one year of methotrexate therapy, the patient showed no meaningful clinical improvement (Fig. 1A–B). Given the severity and resistance to standard therapies, anifrolumab was introduced.

**Fig. 1:**
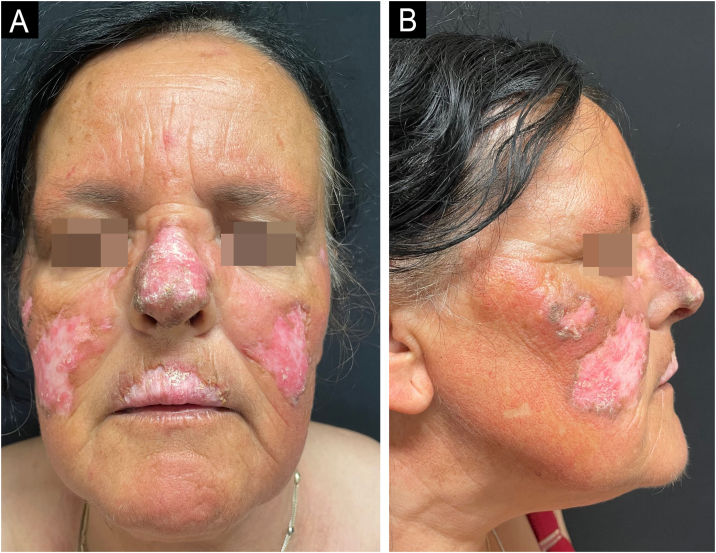
(A‒B) Chronic cutaneous lupus erythematosus of the face before treatment with Anifrolumab.

A remarkable clinical response was observed after only eight weeks of treatment. There was near-complete resolution of facial inflammation and scaling, with noticeable reduction in lesion size and repigmentation at the periphery of previously hypopigmented areas (Fig. 2A–B). No adverse events were reported.

**Fig. 2:**
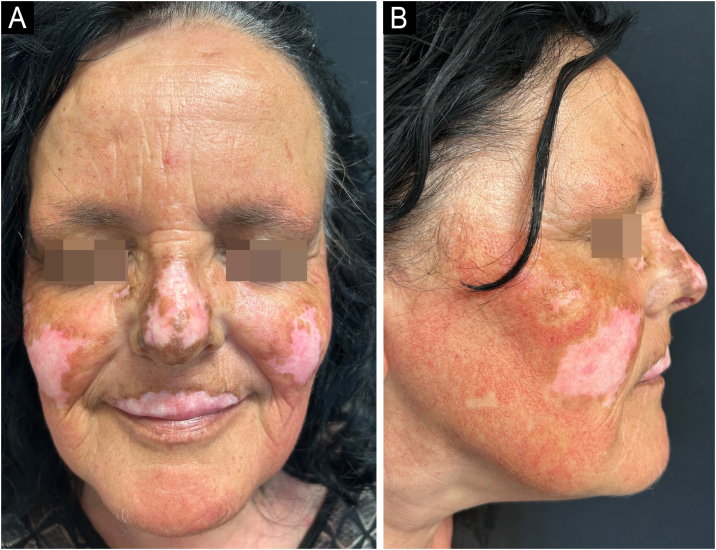
(A‒B) Chronic cutaneous lupus erythematosus of the face after only 8 weeks of treatment with Anifrolumab.

This case highlights a severe, scarring form of DLE that was unresponsive to multiple standard therapies, including hydroxychloroquine, topical and systemic corticosteroids, and methotrexate. Treatment with anifrolumab led to a rapid and substantial clinical improvement, suggesting that type I interferon blockade may be a promising therapeutic option for refractory CLE, particularly in patients with chronic and atrophic lesions and poor quality of life. This case underscores the potential role of anifrolumab in managing difficult-to-treat CLE and supports the need for further studies to establish its efficacy and safety in cutaneous forms of lupus beyond systemic lupus erythematosus.

## ORCID ID

José Ramos: 0009-0007-5362-1141

Hugo Leme: 0009-0008-9011-2618

António Magarreiro-Silva: 0000-0002-0433-0352

## Financial support

None declared.

## Authors’ contributions

José Ramos: Writing of the manuscript or critical review of important intellectual content; Critical review of the literature; Final approval of the final version of the manuscript.

Hugo Leme: Intellectual participation in the propaedeutic and/or therapeutic conduct of the studied cases; Final approval of the final version of the manuscript.

António Magarreiro-Silva: Critical review of the literature; Final approval of the final version of the manuscript.

## Research data availability

Does not apply.

## Conflicts of interest

None declared.
